# Small-cell neuroendocrine carcinoma of the descending colon in a young adult: a case report

**DOI:** 10.3389/fonc.2026.1835901

**Published:** 2026-05-25

**Authors:** Rong-Chao Zhou, Gang Zhou, Zhe Liang

**Affiliations:** 1 Department of Radiology, Tianjin Academy of Traditional Chinese Medicine, Tianjin, China; 2 Department of Radiology, Tianjin Academy of Traditional Chinese Medicine Affiliated Hospital, Tianjin, China; 3 Department of Pathology, Tianjin Academy of Traditional Chinese Medicine Affiliated Hospital, Tianjin, China

**Keywords:** case report, colonic neuroendocrine neoplasms, computed tomography, descending colon, small-cell neuroendocrine carcinoma

## Abstract

Neuroendocrine neoplasms (NENs) are a heterogeneous group of tumors arising from neuroendocrine cells with malignant potential. NENs of the colorectum are uncommon, and colonic neuroendocrine neoplasms (CNENs) are particularly rare. Here, we report the diagnostic and therapeutic course of a man in his 25-year-old with small-cell neuroendocrine carcinoma (SCNEC) of the descending colon. The patient presented with sudden, severe abdominal pain accompanied by nausea and vomiting. Computed tomography (CT) revealed circumferential wall thickening of the descending colon, blurring of the surrounding fat planes, and multiple small regional lymph nodes; contrast-enhanced CT demonstrated marked, homogeneous, and persistent enhancement. The initial imaging findings were easily confounded with inflammatory disease, and the patient declined further evaluation and was discharged. Nine months later, he was readmitted because of progressive intestinal obstruction. Imaging showed substantial progression of the lesion with extension to the sigmoid colon, accompanied by signs of extra-serosal invasion and peritoneal dissemination. Colonoscopy and histopathology confirmed SCNEC. The patient received ascites control, etoposide plus carboplatin chemotherapy, and supportive care, but the response was poor. This combination of young age and tumor location in the descending colon appears to be uncommon in the existing literature. The case is further distinguished by the presence of circumferential colonic wall thickening on imaging, a feature absent in prior reports. This case highlights that colonic SCNEC in young patients may present with atypical imaging features. Colonic NENs should be included in the differential diagnosis, and early endoscopic biopsy should be performed; definitive diagnosis relies on histopathology and immunohistochemistry to facilitate early recognition and standardized management.

## Introduction

Neuroendocrine neoplasms (NENs) are a heterogeneous group of tumors arising from neuroendocrine cells throughout the body and characterized by malignant potential. Colorectal NENs are overall uncommon, although their anatomic distribution varies substantially across populations and studies; in one cohort, rectal and colonic primaries accounted for approximately 49.5% and 6.2% of gastroenteropancreatic NENs (GEP-NENs), respectively (1, 2). Colorectal neuroendocrine carcinomas (NECs) are even rarer, representing about 0.6%–3.9% of all colorectal cancers (3). Small-cell neuroendocrine carcinoma of the colon (SCNECC) is rarer still, with an incidence of <0.2% among all colorectal tumors, and it commonly occurs between 60 and 70 years of age (4).

Most colorectal NENs do not secrete clinically significant hormones and are therefore predominantly nonfunctional tumors (5). Their clinical manifestations are often nonspecific, commonly presenting with hematochezia, changes in bowel habits or defecation discomfort, a sensation of rectal/perianal tenesmus, localized pain, and varying degrees of anemia, which can be easily confused with inflammatory bowel disease, adenocarcinoma, and other intestinal disorders (6). Accordingly, we report a case of small-cell neuroendocrine carcinoma in a young patient, focusing on its atypical imaging features and interpreting these findings in conjunction with pathological evidence, with the aim of providing diagnostic clues and new perspectives for the differential diagnosis of this rare, highly aggressive malignancy in young individuals.

## Case description

A 25-year-old male presented to our hospital with a 2-hour history of sudden, severe abdominal pain accompanied by nausea and vomiting. He reported a history of intermittent mild abdominal pain. He denied hypertension, heart disease, diabetes, cerebrovascular disease, and a history of hepatitis, tuberculosis, malaria, or other infectious diseases. He also denied any family history of hereditary disorders. This case report was prepared in accordance with the CARE guidelines.

Non-contrast abdominal computed tomography (CT) was performed using a Siemens Go Top 64-slice CT scanner which showed circumferential wall thickening of the descending colon, blurring of the surrounding fat planes, and multiple small regional lymph nodes. Contrast-enhanced CT demonstrated marked, homogeneous, and persistent enhancement, with a longitudinal extent of >8 cm in the descending colon. Based on the patient’s age and imaging findings, the initial differential diagnosis included: (1) inflammatory disease and (2) neoplastic disease. Serum tumor markers were as follows: carbohydrate antigen 72-4 (CA72-4) 17.58 IU/mL (reference range: 0–6.9 IU/mL), carcinoembryonic antigen (CEA) 5.410 ng/mL (0–5.0 ng/mL), and carbohydrate antigen 19-9 (CA19-9) 100.97 IU/mL (0–37 IU/mL). Colonoscopy was recommended to establish a definitive diagnosis, given the possibility of a colonic neoplasm and the need for timely evaluation to avoid delayed treatment. However, the patient and his family declined further investigations and treatment due to financial concerns and psychological resistance to a potential cancer diagnosis; he subsequently signed an informed refusal and left the hospital against medical advice (AMA).

Nine months after the initial presentation, the patient developed paroxysmal periumbilical colicky pain without an obvious trigger, which gradually progressed to persistent distending pain with intermittent exacerbations. He also experienced frequent vomiting without symptom relief. Thereafter, he developed obstipation, with cessation of both bowel movements and flatus. He had lost more than 20 kg over the preceding 3 months. He was admitted via the emergency department with a diagnosis of partial intestinal obstruction for further management. Follow-up CT showed marked progression, with more pronounced circumferential thickening of the descending colon, an increased involved segment, and inferior extension to the sigmoid colon. The serosal surface appeared irregular, with increased density of the adjacent fat planes. Scattered regional lymph nodes were noted. Multiple small nodules were present in the peritoneum, omentum, and mesentery, accompanied by massive ascites. These findings suggested a high likelihood of malignancy, prominent extramural invasion, and multiple peritoneal metastatic nodules (**Figure 1**).

**Figure 1:**
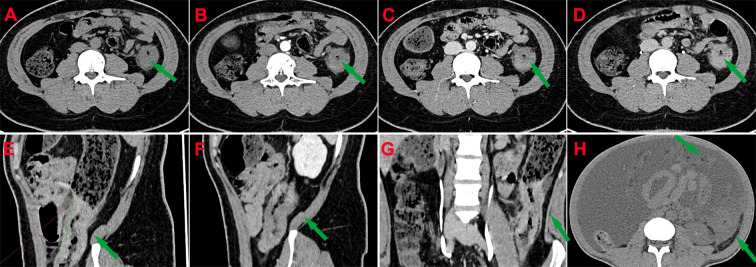
Contrast-enhanced computed tomography (CT) images. **(A)** Non-contrast CT image (40 HU). **(B–D)** Contrast-enhanced CT images, including the arterial phase [**(B)** 43 HU], portal venous phase [**(C)** 61 HU], and delayed phase [**(D)** 85 HU]. **(E, F)** Three-dimensional sagittal reconstructions showing involvement of the descending colon with a length exceeding 8 cm; **(E)** with a scale bar, **(F)** without a scale bar. **(G)** Three-dimensional coronal reconstruction. **(H)** Follow-up CT image obtained 9 months later.

Repeat tumor marker testing revealed: cytokeratin 19 fragment (CYFRA21-1) 8.62 ng/mL (0–3.3 ng/mL), CA72-4 45.60 IU/mL (0–6.9 IU/mL), CEA 145.170 ng/mL (0–5.0 ng/mL), and CA19-9 >1000 IU/mL (0–37 IU/mL). All markers were markedly elevated compared with the first admission. The patient underwent paracentesis with indwelling catheter drainage for ascites control. Colonoscopy revealed severe luminal stenosis in the sigmoid colon at approximately 30 cm from the anal verge, preventing further passage of the endoscope. The sigmoid mucosa was diffusely elevated, and a focal protruding lesion was observed at the stenotic segment; prominent vessels were seen on the surface, and the tissue was friable and prone to bleeding. Histopathological examination was performed on tissue samples, with microscopic images acquired using an Olympus BX46 microscope (Olympus, Tokyo, Japan) equipped with a ×10 objective lens (model: Olympus PlanN). Digital pathological images were captured via the iViewer image acquisition software (version7.2.7.2), and immunohistochemical (IHC) staining images were obtained with the same microscopic imaging system, which confirmed colonic neuroendocrine carcinoma consistent with small-cell type (**Figure 2**). Immunohistochemistry showed CgA (−), CD56 (focally+), Ki-67 (~90%+), CK (+), CD20 (−), CDX-2 (+), CK20 (−), Syn (−), and TTF-1 (−) (**Figure 3**).

**Figure 2:**
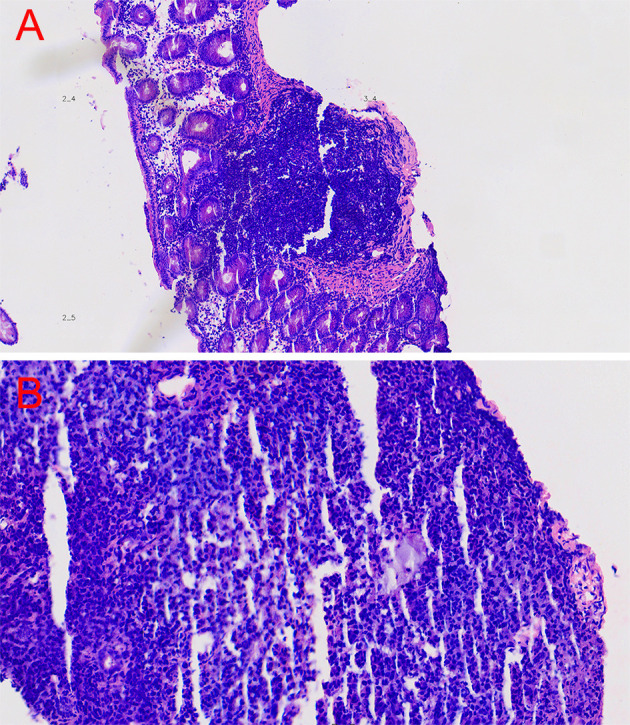
Microscopic examination reveals a dense proliferation of carcinoma cells in a diffuse and cord-like arrangement. **(A)** H&E (×100); **(B)** H&E (×200).

**Figure 3:**
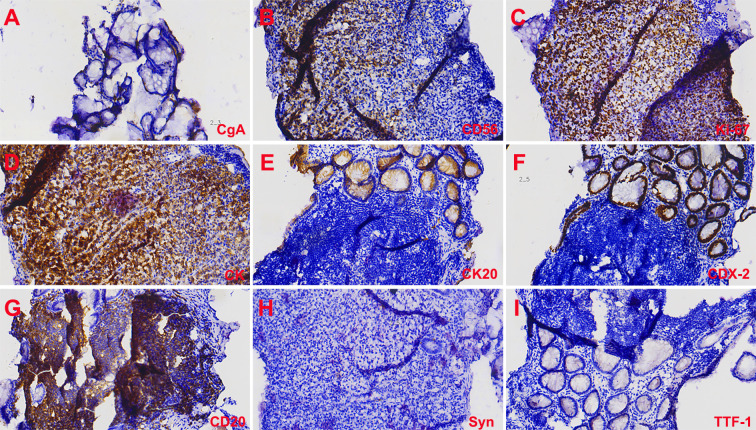
Immunohistochemical (IHC) results of the patient (×100). **(A)** Chromogranin A (CgA): tumor cells show focal cytoplasmic positivity. **(B)** Cluster of Differentiation 56 (CD56): diffuse membranous and cytoplasmic positivity in tumor cells. **(C)** Ki-67 Proliferation Index (Ki-67): high proliferative index, with strong nuclear staining in a large proportion of tumor cells. **(D)** Pan-cytokeratin (CK): diffuse, strong cytoplasmic positivity, supporting epithelial origin. **(E)** Cytokeratin 20 (CK20): negative in tumor cells, with positive staining in adjacent normal glandular epithelium as internal control. **(F)** Caudal Type Homeobox 2 (CDX-2): negative in tumor cells. **(G)** Cluster of Differentiation 20 (CD20): negative in tumor cells, highlighting background lymphocytes. **(H)** Synaptophysin (Syn): negative in tumor cells. **(I)** Thyroid transcription factor-1 (TTF-1): negative in tumor cells.

After a definitive diagnosis of SCNEC with peritoneal metastasis, the patient was administered the first-line chemotherapy regimen for poorly differentiated neuroendocrine carcinoma: carboplatin combined with etoposide (CE regimen). carboplatin (AUC 5-6) was administered intravenously on day 1 of each cycle, and etoposide 100 mg/m² was administered intravenously on days 1–3 of each cycle, with a 3-week interval as one complete chemotherapy cycle. After completing one cycle of chemotherapy, the patient underwent a follow-up abdominal CT scan that demonstrated no improvement in the relevant lesions. In the meantime, the patient’s clinical symptoms showed no signs of relief, and his general physical condition deteriorated markedly, which rendered him unable to tolerate further therapeutic interventions. As a result, the patient was discharged from the hospital.

At the initial presentation, the patient declined further examination due to financial concerns and psychological resistance to a potential cancer diagnosis. After disease progression, he accepted hospitalization and diagnostic procedures. However, due to poor treatment response and worsening condition, he ultimately chose to discontinue further therapy. The overall clinical course of the patient is summarized in (**Figure 4**).

**Figure 4:**
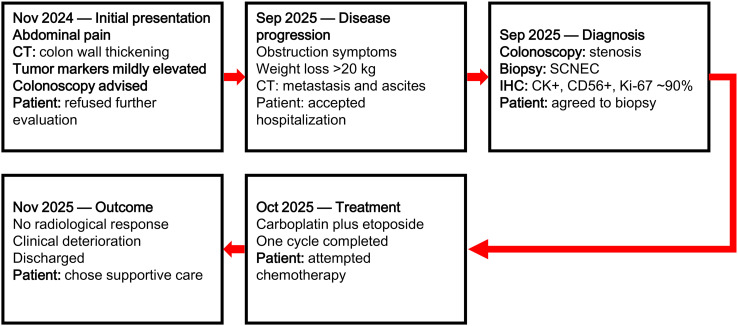
Clinical timeline illustrating the hospital course, diagnostic process, treatment, and outcome of the patient.

## Discussion

Neuroendocrine neoplasms (NENs) can arise anywhere along the gastrointestinal tract, with tumors of the stomach, intestine, and pancreas being relatively common. Malignant NENs account for only approximately 2% of gastrointestinal malignancies, and small-cell neuroendocrine carcinoma (SCNEC) is even rarer, with an incidence of <0.2% among all colorectal tumors. Recent studies have suggested a marked increase in the incidence of colorectal NENs. This trend may reflect a true rise in disease occurrence; however, it may also be attributable to wider use and improved performance of endoscopy and imaging, greater awareness of NENs among clinicians and patients, and increased case ascertainment and data collection (7, 8).

Colorectal neuroendocrine neoplasms (CNENs) most frequently occur in the cecum, followed by the sigmoid colon and ascending colon, and they predominantly affect older adults (>60 years). Clinical manifestations are often nonspecific and may include abdominal pain, changes in bowel habits, an abdominal mass, weight loss, and anemia. Presentations depend on tumor location and size, and some patients seek medical attention because of complications such as bowel obstruction or perforation (9). On CT, CNENs typically appear as eccentric bowel-wall thickening or a mass-like soft-tissue lesion, whereas circumferential, symmetric wall thickening is relatively uncommon. These tumors are usually hypervascular; contrast-enhanced CT often shows moderate to marked enhancement, frequently in a progressive pattern. Necrosis is relatively uncommon, but enhancement is often heterogeneous (10). Serosal irregularity and blurring with increased attenuation of adjacent fat planes suggest varying degrees of extramural invasion. Regional lymph node metastasis is relatively frequent, and metastatic nodes often demonstrate moderate to marked enhancement on contrast studies.

In the present case, the patient was a man in his 25-year-old with a tumor located in the descending colon. He initially presented with abdominal pain and distension, and developed intestinal obstruction as the disease progressed. Imaging showed circumferential wall thickening of the descending colon with an involved length >8.5 cm and marked, homogeneous, and persistent enhancement on contrast-enhanced CT. The surrounding fat planes were indistinct, and multiple regional lymph nodes were present, without definite imaging evidence of distant metastasis at that time. Because these findings overlap with those of inflammatory disease, imaging-based differentiation was challenging, and definitive diagnosis ultimately relied on histopathology and immunohistochemistry.

The differential diagnosis in this case mainly included colorectal adenocarcinoma and lymphoma. Conventional colorectal adenocarcinoma often presents as a focal mass-like lesion, whereas the present tumor showed predominantly circumferential wall thickening without forming a discrete mass, even at an advanced stage, which was less typical (11, 12). Lymphoma was also considered; however, the imaging and endoscopic findings were not fully consistent with its usual presentation. Recent evidence has also emphasized that colorectal small-cell/neuroendocrine carcinoma may show endoscopic–histologic discordance because of predominant submucosal growth, and its differential diagnosis includes poorly differentiated adenocarcinoma and lymphoma (13). Therefore, the final diagnosis in this case depended on histopathological examination combined with immunohistochemical findings. Taken together with the existing literature, these findings suggest that although colorectal neuroendocrine carcinomas typically present as focal or asymmetric lesions, atypical patterns such as circumferential wall thickening may occur and should not be overlooked in clinical practice.

Neuroendocrine differentiation in poorly differentiated neuroendocrine carcinomas may not always be reflected by classical immunohistochemical markers. According to the WHO Classification of Tumours of the Digestive System (5th edition), the expression of synaptophysin and chromogranin A in high-grade neuroendocrine carcinomas can be focal, weak, or even completely absent (14). In contrast, CD56 is more frequently expressed, although it lacks specificity. In the present case, despite negative staining for synaptophysin and chromogranin A, the diagnosis was supported by characteristic morphological features, a high proliferative index (Ki-67 ~90%), and focal CD56 positivity. Similar immunohistochemical profiles have been reported in colorectal neuroendocrine carcinomas in previous studies (15–17). However, the absence of additional neuroendocrine markers such as INSM1 represents a limitation of this study.

Classification of NENs follows the World Health Organization (WHO) nomenclature and grading system (18). Based on differentiation, NENs are categorized as well-differentiated neuroendocrine tumors (NETs) or poorly differentiated neuroendocrine carcinomas (NECs). Well-differentiated NETs are further graded as G1, G2, or G3 according to mitotic count and the Ki-67 proliferation index. NECs are subdivided into large-cell neuroendocrine carcinoma (LCNEC) and small-cell neuroendocrine carcinoma (SCNEC). Currently, in high-grade NENs, the distinction between G3 NET and poorly differentiated NEC largely depends on morphological and histopathological assessment. Importantly, G3 NET differs substantially from NEC in clinicopathologic features, treatment strategies, and prognosis; therefore, these entities should be distinguished to guide management appropriately (19). Ki-67 is a proliferation-associated antigen detectable in the nuclei of actively dividing cells (20), and its level is closely related to NEN grade and prognosis. In this case, the Ki-67 labeling index was approximately 90%, indicating highly proliferative, aggressive biology with a propensity for rapid progression and distant metastasis, consistent with the clinical course.

Poorly differentiated gastrointestinal neuroendocrine carcinomas (GI-NECs) are rare but highly malignant and associated with poor outcomes. Early recognition and definitive diagnosis facilitate standardized treatment, thereby improving quality of life and potentially prolonging survival. For localized CNENs, surgical resection is the primary treatment, including local excision and radical resection. For colorectal NENs ≤2.0 cm confined to the mucosa/submucosa without regional lymph node or distant metastasis, endoscopic submucosal dissection (ESD) may be considered (21). In contrast, colonic NEC and colonic NETs (CNETs) ≥2 cm are generally managed with radical resection (22). For CNENs with distant metastasis, however, there is no consensus regarding the role and extent of surgery.

With respect to systemic therapy, platinum-based chemotherapy (carboplatin or cisplatin) combined with etoposide for 4–6 cycles is the recommended first-line regimen for poorly differentiated NEC. This regimen has been shown to improve overall outcomes in intestinal poorly differentiated NENs, extending median overall survival to approximately 8–20 months (23). Therefore, for patients with metastatic colonic neuroendocrine carcinoma (CNEC), clinicians should comprehensively evaluate differentiation and grade, the primary site, extent of invasion, and overall condition, and formulate an individualized treatment strategy based on multidisciplinary team (MDT) discussion.

## Conclusions

Small-cell neuroendocrine carcinoma (SCNEC) is extremely rare and is typically poorly differentiated, highly aggressive, and prone to regional lymph node and distant metastases. When similar findings are encountered in clinical and radiologic evaluation, colonic neuroendocrine neoplasms should be included in the differential diagnosis, and early endoscopic biopsy should be pursued. Definitive diagnosis still relies on histopathology and immunohistochemistry. Early and accurate diagnosis facilitates multidisciplinary assessment and individualized treatment planning, thereby improving therapeutic effectiveness and potentially patient outcomes. Furthermore, This case further underscores the importance of clinicians’ astute judgment and high vigilance for malignancies in young patients. In addition, effective patient communication and psychological support are equally critical to improving treatment adherence and prognosis.

## Data Availability

The original contributions presented in the study are included in the article/supplementary material. Further inquiries can be directed to the corresponding author.
